# Colorectal tumor prevention by the progestin medroxyprogesterone acetate is critically dependent on postmenopausal status

**DOI:** 10.18632/oncotarget.25703

**Published:** 2018-07-17

**Authors:** Bartolomeus J. Meijer, Mattheus C.B. Wielenga, Patricia B. Hoyer, James M. Amos-Landgraf, Theodorus B.M. Hakvoort, Vanesa Muncan, Jarom Heijmans, Gijs R. van den Brink

**Affiliations:** ^1^ Tytgat Institute for Liver and Intestinal Research and Department of Gastroenterology and Hepatology, Academic Medical Center, Amsterdam, The Netherlands; ^2^ Department of Internal Medicine, Academic Medical Center, Amsterdam, The Netherlands; ^3^ Department of Physiology, University of Arizona, Tucson, AZ, USA; ^4^ Department of Veterinary Pathobiology, University of Missouri, Columbia, MO, USA; ^5^ GlaxoSmithKline, Medicines Research Center, Stevenage, UK

**Keywords:** colon cancer, chemoprevention, animal models, menopause, hormone replacement

## Abstract

The large randomized placebo controlled trials of the Women’s Health Initiative have shown that the combination of estrogen and progestin medroxyprogesterone acetate (MPA) protects from colorectal cancer in postmenopausal women. No effect was observed in women treated with estrogen alone. This suggests that progesterone, or more specifically the progestin MPA may have chemopreventive activity. The effect of MPA on colorectal carcinogenesis has been difficult to study in animal models. Most models are not affected by either depleting female hormones by ovariectomy or treatment with MPA. Importantly, an ovariectomy fails to reproduce one of the hall marks of the postmenopausal state in women with intact ovaries. That is, the continued production of androgens by the atrophic postmenopausal ovaries. Here we show that adenoma incidence is increased in the vinyl cylcohexene diepoxide (VCD) mouse model of the menopause compared to age matched fertile female mice. Treatment with MPA protected VCD treated mice from adenomagenesis, but had no effect on adenoma numbers in age-matched fertile female mice. Our data show that the protective effect of MPA depends on the postmenopausal state and suggest that MPA monotherapy may be studied as a chemopreventive agent in postmenopausal women.

## INTRODUCTION

Male sex is one of the strongest risk factors for the development of colorectal cancer development. The incidence of both colorectal adenoma and cancer development is higher in males than females [1, 2]. This suggests that the process of colorectal carcinogenesis is influenced by sex hormones. The male predominance of CRC development could be driven either by a protective effect of female hormones or a tumor promoting effect of male hormones. We have previously identified two animal models in which males developed more colorectal adenomas than females. In both models, we found that sex disparity in colonic adenomagenesis is driven by tumor promotion by male hormones, not protection by female hormones [3]. This suggests that sex disparity in CRC incidence is driven by male hormones. The observation that male hormones may be driving colorectal carcinogenesis is at apparent contradiction with results from the randomized studies of the Women’s Health Initiative. In these very large randomized controlled studies, women with an intact uterus received either placebo or a combination of equine estrogen (E2) and medroxyprogesterone acetate (MPA), whereas women who underwent hysterectomy received placebo or E2 alone. The studies showed that E2/MPA combination therapy substantially reduced the risk of CRC development [4, 5] whereas treatment with E2 alone had no effect [6]. Together these studies suggest that MPA or the MPA/E2 combination therapy protect against the development of CRC. Animal models of CRC development have failed to consistently show a protective effect of female hormones on CRC. In our own experiments we have not observed an effect of either treatment with MPA or deletion of the progesterone receptor in rodent models of adenoma development. However, experiments performed by us and others had an important caveat since they relied on ovariectomy for elucidating the effect of female hormones. Although this technique reliably removes the source of female hormones, it fails to reproduce important aspects of the biology of the menopause. Human ovaries in postmenopausal women do not produce female sex hormones, however, they are still hormonally active and produce substantial amounts of androgens [7, 8]. The abundance of androgens together with decreased levels of sex hormone binding globulin in the circulation during menopause [9] provides an increased biological availability of androgens. Therefore we set out to investigate the chemopreventive effects of MPA in an adenoma development mouse model combined with a model for menopause.

## RESULTS

**Figure 1:**
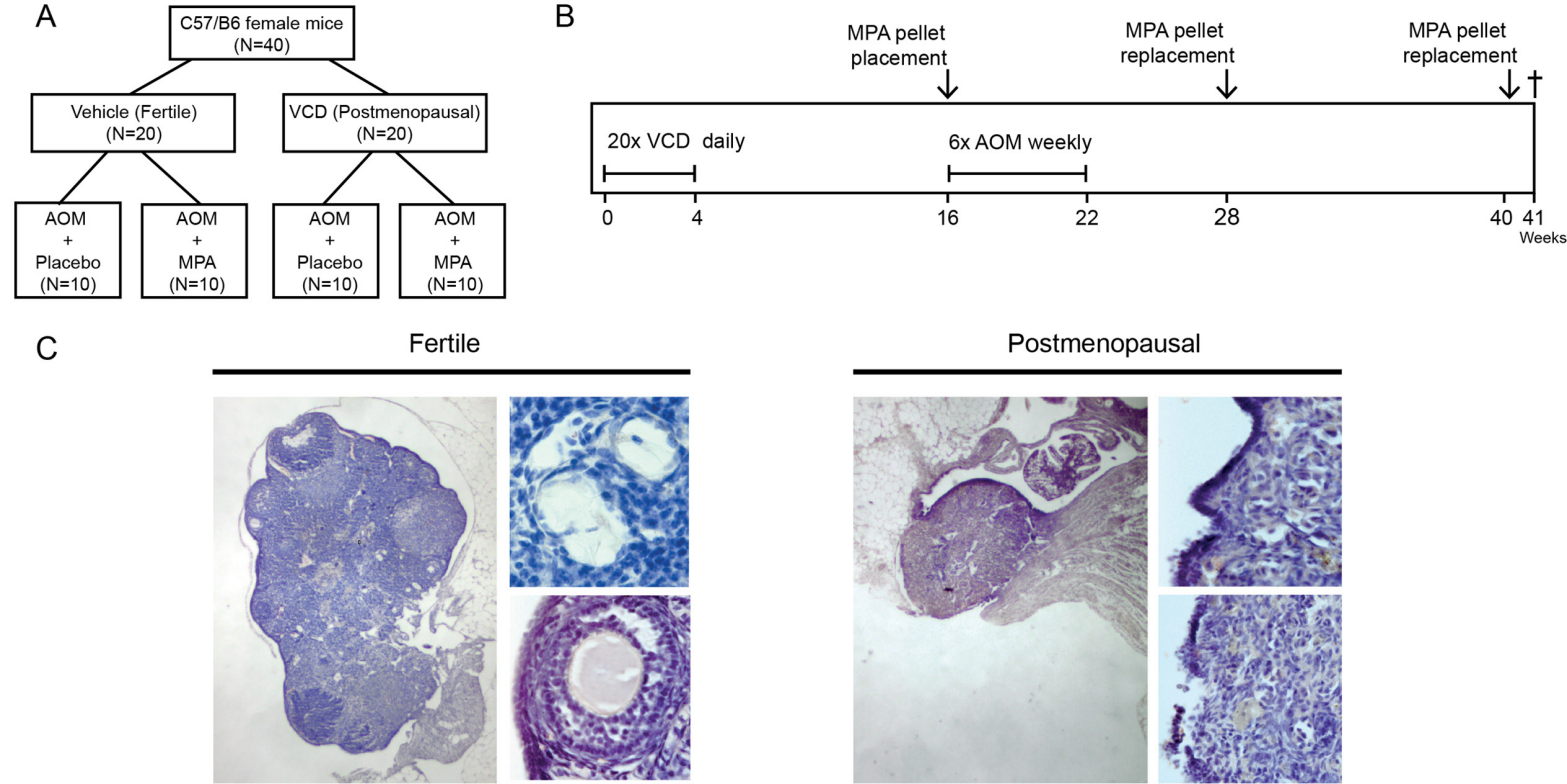
Experimental setup and VCD induced follicle depleted ovaria **(A)** Mice were allocated to one of the four treatment groups (*n=10 per group)* **(B)** At six weeks of age mice were injected intraperitoneally in 21 subsequent days with either 4-vinylcyclohexene diepoxide (VCD, 160 mg/kg in corn oil) to induce postmenopausal state or vehicle only according as described previously (Hoyer et al) [9]. Three months after VCD treatment mice were 6 times injected with AOM (10mg/kg in 0.9%NaCl) weekly. Simultaneously with the AOM, hormonal replacement therapy was started by placing medroxy-progesteron acetate (MPA, 7,5mg in 90days) or vehicle slow release pellets subcutaneously. Pellets were replaced with new pellets after 12 and 24 weeks. 30 weeks after start AOM mice were sacrificed. **(C)** *H&E* Representative ovary of a fertile mouse (left) showing presence of follicles in all stages. Top (40X magnification) primary follicle with single layer of cuboidal cells. Bottom (20X magnification) secondary follicle with multiple layers of granulosa cells. Right panel shows a representative ovary of a VCD treated postmenopausal mouse depleted of follicles.

We used the 4-vinylcyclohexene diepoxide (VCD) to obtain a model for menopause in mice [10, 11], hereinafter referred to as postmenopausal state. In this model, daily injections with VCD leads to ovarian failure as the result of follicle depletion. Ovarian exhaustion is induced after approximately 50 days. Age matched fertile female mice that had received daily vehicle (corn oil) injections were used as controls. We subsequently induced colonic adenoma formation using six injections with the carcinogen azoxymethane (AOM) [12]. In all mice, 90-day slow release pellets were implanted subcutaneously containing either 7,5 mg medroxyprogesterone (MPA) or placebo (Figure 1A, 1B). This dose was tenfold higher than the IC50 dose that resulted in ovulation inhibition in rats (1mg/ kg/day), allosterically converted between rats and mice (4mg/kg/day) [13, 14]. Mice were sacrificed at 47 weeks of age (Figure 1A). In VCD treated mice, ovarian histology confirmed atrophy and depletion of ovarian follicles (Figure 1C), as is similarly observed in postmenopausal women.

**Figure 2:**
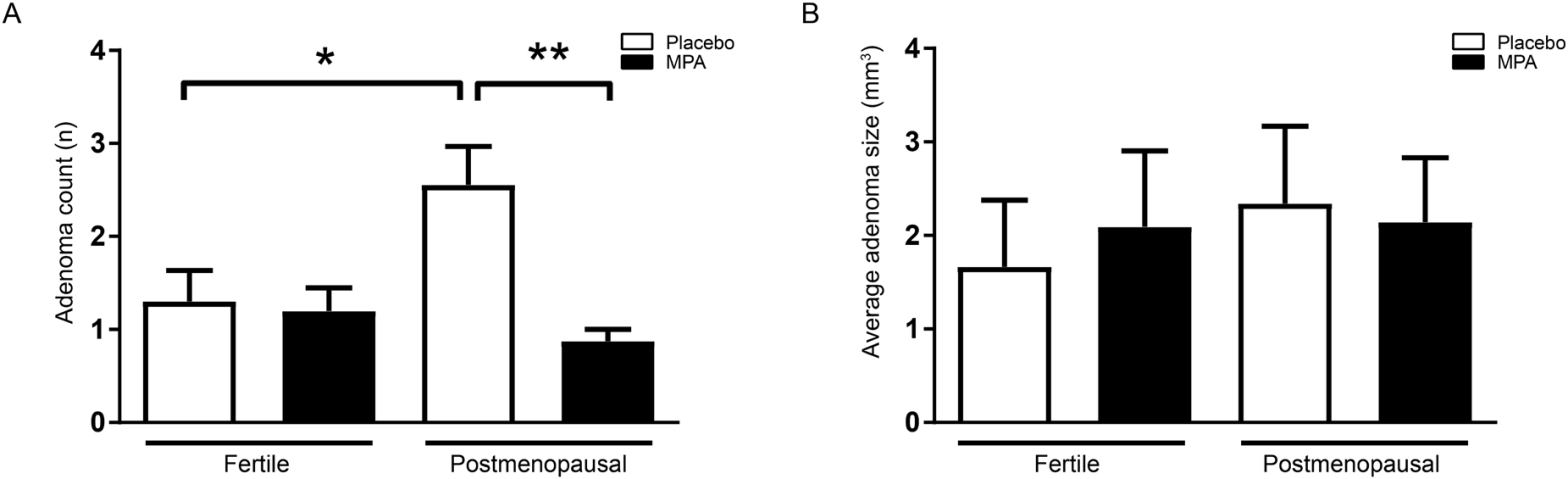
MPA protects from adenoma formation in VCD treated mice **(A)** Total adenoma number in fertile mice colon receiving placebo pellets (n=10), fertile mice receiving MPA pellets (n=10), VCD treated postmenopausal mice receiving placebo pellets (n=9) and VCD treated postmenopausal mice receiving MPA pellets (n=8). **(B)** Average adenoma size in colon per mouse, measured using a ruler guide. One-way analysis of variance (ANOVA) test was used, followed by a Bonferroni post-test for multiple comparisons. Data are mean ± SEM (^*^= p-value (< 0,05),^**^= (^*^= p-value (< *0,01).*

**Figure 3:**
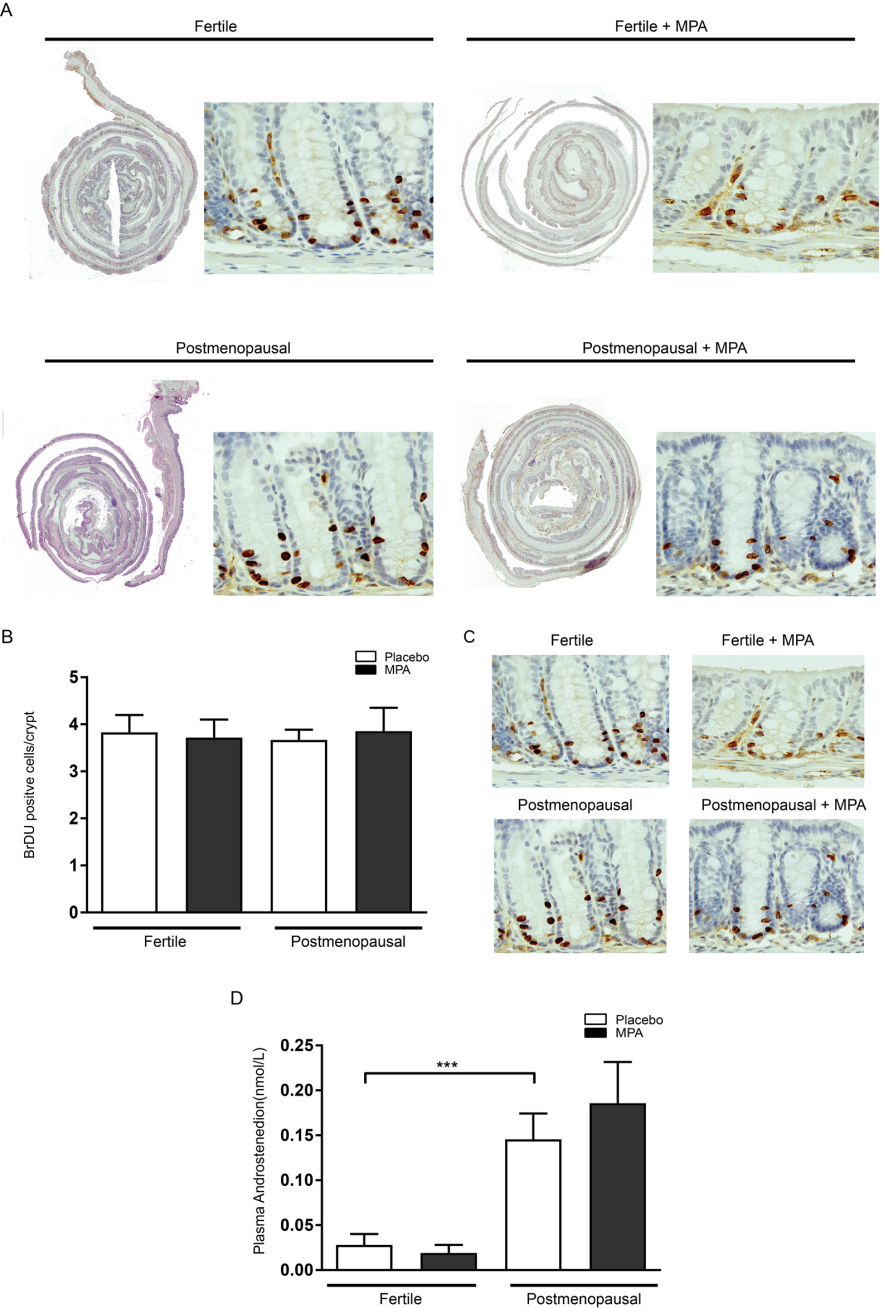
Epithelial proliferation is not influenced by menopause induction and MPA treatment **(A)** One hour prior to sacrifice, all animals were injected with 200 μl BrdU (10 mg/ml in PBS. Colonic tissues were embedded as swiss rolls, deparaffinised in xylene and rehydrated. **(B)** Quantification of BrdU positive cells per crypt for all treatment groups. **(C)** *Unaltered Wnt signalling between treatment groups.* B-catenin staining on colonic tissue. **(D)** *Menopause induction increases circulating levels of serum androstenedione.* Serum levels of circulating androstenedione for all treatment groups. One-way analysis of variance (ANOVA) test was used, followed by a Bonferroni post-test for multiple comparisons. Data are mean ± SEM (^*^= p-value (< 0,05),^**^= (^*^= p-value (< *0,01).*

In control animals (fertile females that had received placebo pellets), AOM injections resulted in an incidence of colonic adenoma of 80% with an average multiplicity of 1.3, corresponding to previous literature [12, 15-16]. Long term treatment of fertile females with MPA did not alter adenoma number or size in these mice (Figure 2A, 2B). These results confirmed previous experiments reporting no effect of MPA on adenoma precursor lesions (aberrant crypt foci) or adenomas in ovariectomized rats [3, 17]. In postmenopausal mice that had received placebo pellets, the incidence of adenoma was 100% and multiplicity was significantly increased compared to untreated fertile mice (2.6 vs 1.3 *P* < 0.05). Surprisingly, in postmenopausal mice that had received MPA, colonic adenoma numbers had normalized to the level found in fertile mice (*VCD-MPA:* 0.9 vs *VCD-placebo*: 2.6, *P* < 0.001) (Figure 2A). Although postmenopausal state and treatment with MPA influenced tumor incidence, none of the conditions affected average tumor size (Figure 2B). To further examine the effect of menopause and MPA treatment on adenomagenesis, we assessed epithelial proliferation by quantification of short term BrdU incorporation in non-adenomatous colonic mucosa. We did not observe altered proliferation comparing different treatment groups (Figure 3A, 3B). Colorectal adenomas most frequently develop as the result of hyperactivation of the oncogenic Wnt pathway. The hallmark of activated Wnt signaling is nuclear accumulation of β-catenin. We did not observe gross differences in the nuclear accumulation of β-catenin in colon of different treatment groups (Figure 3C). Together, these data are compatible with the observation that hormonal status did not influence adenoma size. Our data suggest that menopausal status and MPA specifically influence tumor initiation but not growth.

**Figure 4:**
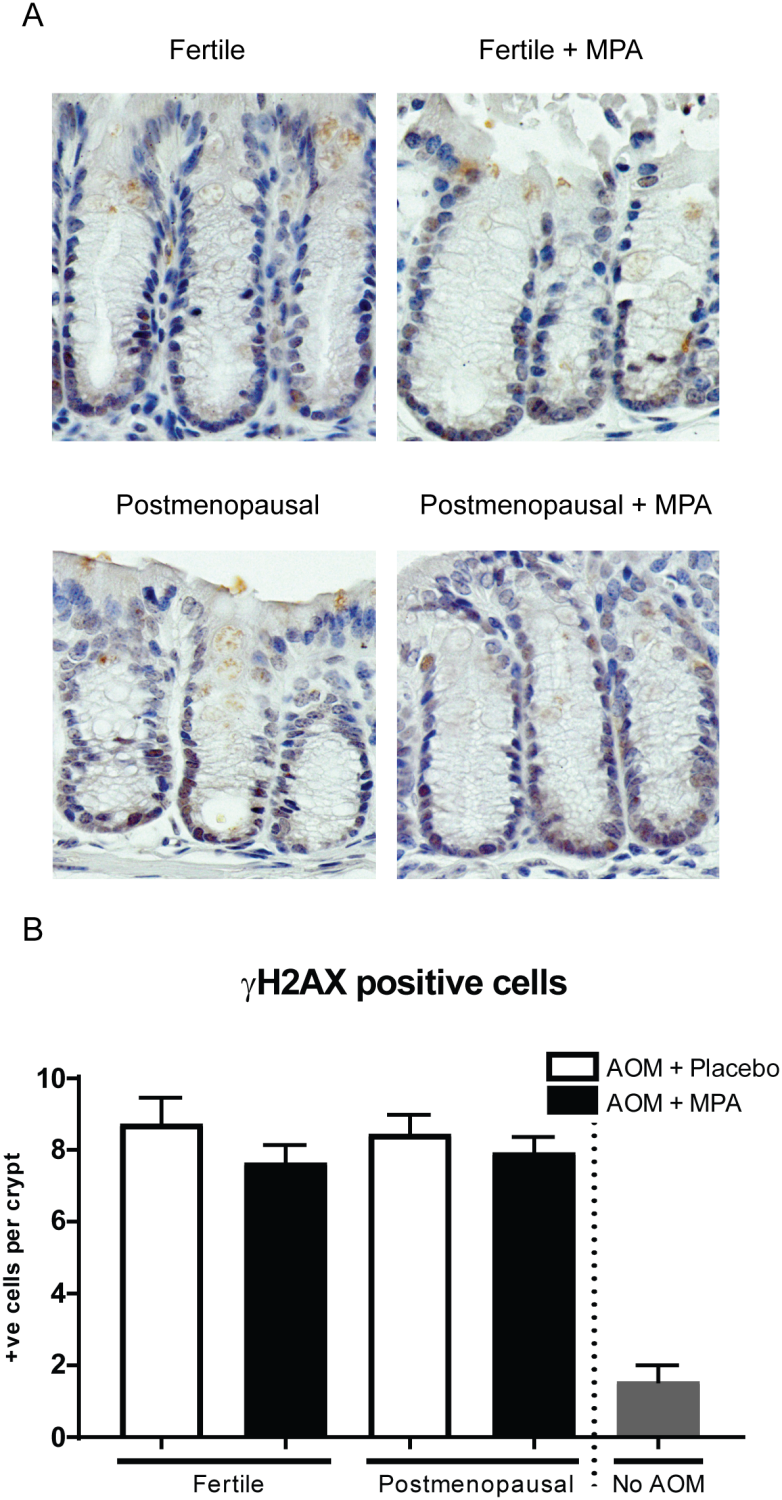
MPA treatment does not rescue increased DNA damage upon azomxymethane treatment shown by γ-H2AX staining **(A)** γ-H2AX staining on colonic tissue 30 weeks after start AOM **(B)** Quantification of γ-H2AX positive cells per colonic crypt. Dotted line separates experiment mice (*n=10)* from mice not treated with AOM as control (*n=3), per mouse approximately 20 crypts were count.* One-way analysis of variance (ANOVA) test was used, followed by a Bonferroni post-test for multiple comparisons. Data are mean ± SEM (^*^= p-value (< 0,05),^**^= (^*^= p-value (< *0,01).*

Azoxymethane methylates DNA in the intestine and causes DNA double-strand breaks. To examine if the different treatments affected DNA damage, we quantified phosphorylated histone 2AX (γ-H2AX), an established marker for DNA double-strand breaks. The γ-H2AX positive stained cells were mainly located at the basal part of the colon crypts as described (Figure 4) [18]. The number of γ-H2AX positive cells was substantially increased compared to AOM untreated mice. However, the was no difference between the treatment groups, indicating that hormone status did not affect the DNA damage response.

Postmenopausal ovaria exhibit increased androgen production [8], and this is reproduced in the VCD mouse model of the menopause [11]. As we have previously demonstrated that androgens are a tumor promoting factor in animal models of adenomagenesis including the AOM model in this study, we examined if menopausal status and MPA treatment influenced the level of androgen production in the ovaries in our experiment. We thus measured levels of the androgen androstenedione in our experimental groups. Indeed, VCD treated mice had significantly higher levels of serum androstenedione compared to fertile mice (0.024 vs 0.163 nmol/L, p<0.0001). MPA treatment however did not affect androgen levels (Figure 3D).

## DISCUSSION

Our data show that using the VCD model of the menopause in age matched mice, provides an opportunity to understand the influence of postmenopausal status on the risk of CRC. The model may help to unravel the factors that predispose women to CRC after the menopause and the way this risk is influenced by therapeutic intervention such as hormone replacement therapy. Experiments that have examined the role of female hormones in CRC development have relied on using ovariectomies. Ovariectomies are a useful way to study the effect of acute depletion of all sex hormones in the premenopausal state. However, the intervention does not adequately recapitulate the biology of the post-menopausal state where ovaries remain an important source of androgens. This may explain why previous research has failed to demonstrate protective effects of hormone replacement therapy on adenomagenesis [3, 17]. In the model used for this study, postmenopausal mice developed increased colonic adenomas compared to their fertile counterparts. Postmenopausal ovaries increase their androgen production. As we have previously shown that androgens promote adenomagenesis and explain the biological sex difference in adenoma incidence in two rodent models in which such disparity was observed [3], the drop in female sex hormone production and increased androgen secretion may explain increased tumorigenesis.

We find that MPA reduces tumorigenesis in postmenopausal mice, but not in fertile mice. This may be related to an unique property of MPA that is unrelated to its progestagenic activity. MPA has been described as an AR-agonist harboring either androgenic or anti-androgenic effects, dependent on dose and context. MPA binds with a similar affinity to AR as the native ligand 5alfadihydrotestosterone and can disrupt AR signaling [19–21]. However, while a role for the AR in colorectal cancer has been proposed [22], we have previously shown that the AR is not expressed in colonic epithelial cells [3]. We have pursued the hypothesis that the menopausal state and MPA treatment affects adenoma development indirectly via effects on the liver. However, in an experiment in which we examined the effect of these different interventions on the liver, we found no major effects of either VCD treatment or treatment with MPA on liver gene expression using gene expression arrays (data not shown). Alternatively, the effects may be mediated more centrally, for example through effects on the hypothalamus and/ or pituitary. This is a hypothesis that may be pursued in future experiments. Taken together, our work shows that postmenopausal status may specifically increase the risk of CRC and that MPA reduces this risk specifically in a model of the post-menopausal state. Our data suggest that MPA monotherapy may be a chemopreventive strategy to reduce the risk of CRC in postmenopausal women.

## MATERIALS AND METHODS

*Experiments* were performed on C57B6/JOlaHsd wildtype (Harlan laboratories) at six weeks of age. The mice were housed at the animal facilities of the Amsterdam Medical Center, and experiments were performed with consent from the animal ethics committee of the University of Amsterdam (permit number ALC102969). At six weeks of age were injected intraperitoneally in 21 subsequent days with either 4-vinylcyclohexene diepoxide (VCD, 160 mg/kg in corn oil) to induce postmenopausal state or vehicle only according as described previously (Hoyer et al) [9] (n=10 per treatment group). Three months after VCD treatment mice were 6 times injected with Azoxymethane (AOM, 10mg/kg in 0.9%NaCl, Sigma-Aldrich) weekly. Simultaneously with the AOM, hormonal replacement therapy was started by placing medroxy-progesteron acetate (MPA, 7,5mg in 90days, Innovative Research of America) or vehicle slow release pellets subcutaneously. Pellets were replaced with new pellets after 12 and 24 weeks. 30 weeks after start AOM mice were sacrificed.

*For immunohistology* tissue was fixed in 4% ice-cold formalin and embedded in paraffin. Sections of 4 μm were deparaffinised in xylene and rehydrated. H&E staining was performed. *For epithelial proliferation*, BrdU assay was performed. One hour prior to sacrifice, all animals were injected with 200 μl BrdU (10 mg/ml in PBS; Sigma–Aldrich). Tissue was fixed as described above. Colonic tissues were embedded as swiss rolls, deparaffinised in xylene and rehydrated. Endogenous peroxidase was blocked using 0,3% H2o2 in methanol. Sections ere cooked in 0.01M citrate buffer pH 6.0 for 20minutes and incubated with mouse monoclonal anti-BrdU in PBS with 1% BSA and 0.1% Triton-X-100. and then stained with anti-BrdU (clone BMC9318, Roche). Antibody binding was visualized with Powervision horseradish peroxidase-labelled secondary antibodies, and diaminobenzidine for substrate development. All sections were counterstained with Mayer’s haematoxylin.

*B-catenin and γ-H2AX, staining* on colonic tissues was performed with mouse purifiedanti-Beta-catenin (Biosciences Pharmingen) and γ-H2AX (Abcam, ab11174) used as antibody.

*Androstenedion measurement*. Blood was obtained with cardiac puncture during sacrifice. Blood was spinned down and serum was collected and analysed for circulating androstenedione for all treatment groups.

*Adenoma Count* Tumors were macroscopically assessed after ice-cold formalin fixation, by two blinded researchers. Average adenoma size in colon per mouse, was measured using a ruler guide.

### Statistics analysis

Overall, One-way analysis of variance (ANOVA) test was used, followed by a Bonferroni post-test for multiple comparisons. Data are mean ± SEM (^*^= p-value (< 0,05),^**^= (^*^= p-value (< *0,01).*

## Abbreviations

AOM: Azoxymethane
AR: Androgen receptor
CRC: Colorectal cancer
BrdU: Bromodeoxyuridine
E2: Equine Estrogen
MPA: Medroxyprogesterone acetate
PMP: Post-menopausal
VCD: 4-vinylcyclohexene diepoxide
WHI: Women’s Health Initiative
